# Stereotactic radiosurgery in the treatment of essential tremor – a systematic review

**DOI:** 10.3389/fneur.2024.1370091

**Published:** 2024-04-03

**Authors:** Mateusz Bilski, Katarzyna Szklener, Sebastian Szklener, Anna Rudzińska, Natalia Kluz, Jakub Klas, Anna Rodzajewska, Weronika Kuryło, Mateusz Korga, Izabela Baranowska, Sławomir Mańdziuk

**Affiliations:** ^1^Department of Radiotherapy, Medical University of Lublin, Lublin, Poland; ^2^Brachytherapy Department, Saint John’s Cancer Center, Lublin, Poland; ^3^Radiotherapy Department, Saint John’s Cancer Center, Lublin, Poland; ^4^Department of Clinical Oncology and Chemotherapy, Medical University of Lublin, Lublin, Poland; ^5^Hope Clinic Medical Center, Lublin, Poland; ^6^Student Scientific Circle at the Department of Radiotherapy, Medical University of Lublin, Lublin, Poland; ^7^Department of Medical Physics, Saint John’s Cancer Center, Lublin, Poland; ^8^Department of Neurosurgery, Medical University of Lublin, Lublin, Poland

**Keywords:** radiosurgery, tremor, essential tremor, tremor treatment, stereotactic radiotherapy

## Abstract

**Introduction:**

Essential tremor (ET) is the most common movement disorder in adults, with an estimated incidence of up to 1% of the population and 5% of people older than 65 years of age. ET is manifested primarily by bilateral postural and kinetic tremor of the upper limbs with or without neurological symptoms and cognitive deficits. ET disrupts daily tasks and significantly lowers quality of life. Currently available medications alone are often insufficient to control severe symptoms. Several surgical treatment options are available, including stereotactic radiosurgery (SRS)—a minimally invasive treatment option aimed at relieving and controlling tremors.

**Methods:**

We conducted a systematic review of the scientific literature on the use of SRS in the treatment of ET using PubMed, Scopus, Web of Science, Cochrane, ScienceDirect, and ClinicalTrials.gov registry and adhered to the PRISMA guidelines.

**Results:**

The results obtained confirm the high efficacy and safety of the SRS procedure in treating drug-resistant intention tremor. The study results present high response rate reaching 80% and achievement of manual task improvement, lessening of the tremor and increase in the quality of life of the majority of the operated patients. The method also stands out for its favorable balance between efficiency and cost.

**Disscusion:**

Stereotactic radiosurgery is a favourable, safe, efficient and cost-effective method in treatment of the essential tremor. Ongoing research is crucial to refine patient selection criteria for this procedure and further improve the effectiveness of the technique.

## 1 Introduction

Essential tremor (ET) is the most common movement disorder in adults, with an estimated incidence of up to 1% of the population and 5% of people older than 65 years of age (1). The Movement Disorder Society (MDS) defines ET as isolated tremor syndrome of bilateral upper limb action tremor for at least 3 years with or without tremor in other locations (e.g., head, voice, or lower limbs) and absence of other neurological signs, such as dystonia, ataxia, or parkinsonism (2). Familial, otherwise inherited, form of essential tremor constitutes of approximately 50% of cases. ET is manifested primarily by bilateral postural and kinetic tremor of the upper limbs with or without neurological symptoms, cognitive deficits, with some patients experiencing tremors of the head, neck or lower limbs, face, and vocal cords (3–6). Patients report difficulties with daily activities including eating, drinking, dressing, and writing. ET disrupts daily tasks and causes mental distress (1).

Patients diagnosed with ET usually initiate pharmacological therapy with primidone, propranolol, or topiramate. The safety profile, patients’ preferences, and confirmed efficacy are established as a first-line treatment in clinical practice. However, as the improvement rate of the pharmacotherapy is estimated for 50%, medications alone are often insufficient to control severe symptoms (7). To patients with severe spontaneous tremor, who do not achieve treatment response, several surgical options are available (8, 9).

Currently, there are four effective methods of surgical and radiotherapy treatment of patients with ET: deep brain stimulation (DBS), stereotactic radiosrugery (SRS), radiofrequency thalamotomy (RF), and focused ultrasound thalamotomy (FUS).

Stereotactic radiosurgery (SRS) is a minimally invasive treatment option aimed at relieving and controlling tremors. While the standard intervention remains deep brain stimulation, over the past 20 years, ventral intermediate thalamic nucleus (Vim), thalamotomy performed with SRS, has proven to be safe and effective (10). Radiosurgery becomes particularly important in patients with contraindications to surgery or who do not consent to surgical intervention (10). During SRS with GK Vim thalamotomy, patients are placed in a stereotactic frame under local anesthesia. Typically, target area is given a single central maximum dose of 130–152 Gy using a 4-mm collimator. Optimal planning minimizes the radiation exposure of the inner capsule. Modern SRS technique requires no drill holes or cranial electrode puncture and is a relatively non-invasive procedure (11).

## 2 Materials and methods

We conducted a systematic review according to the Population, Intervention, Control, Outcome, Study Design (PICOS) method, which is shown in Table 1. We followed the PRISMA 2020 (Preferred Reporting Items for Systematic Reviews and Meta-Analyses) statement. We searched five databases which were PubMed, Scopus, Web of Science, Cochrane, ScienceDirect, and ClinicalTrials.gov registry. Additional evaluation was conducted via citation searching from selected articles. Two blinded authors independently performed searches using the keywords: (stereotactic radiosurgery or stereotactic radiotherapy or radiosurgery or SRS) AND essential tremor. We identified potential studies and exported them to a reference management program (Mendeley Desktop) for inclusion based on title and abstract and then the full article. The research involved an analysis of all studies published up to 30 November 2023. Although we considered ET cases only, in multiple studies, the only results available included groups of mixed tremor origin, such as Parkinson disease and multiple sclerosis tremor. These results were considered in our analysis and marked accordingly in the summary table below.

**Table 1:** Study design according to the population, intervention, control, outcome, and study design (PICOS) method.

Population	Patients treated with stereotactic radiotherapy for essential tremor (ET).
Intervention	SRS (single fraction ≥5 Gy).
Control	Not applicable (the data will be pooled from single arm trials)
Outcome	Primary: complete response rate, partial response rate, time to response Secondary: acute toxicity, quality of life (QoL), late toxicity, symptoms relapse rate.
Study design	Any retrospective or prospective original studies describing clinical outcomes of patients treated with SRS for ET

### 2.1 Selection criteria

The inclusion criteria were as follows: (1) retrospective and prospective clinical trials with published results and (2) studies published in the English language.

Exclusion criteria were as follows: (1) lack of access to the full text of the manuscript, (2) studies without results and unclear results (3) case reports, (4) review studies, and (5) study protocols.

### 2.2 Data extraction

The extracted data consisted of the author, type of study, sample size, radiotherapy modality, target definition criteria, radiotherapy dose, dose constraints to organs at risk (OARs), time from SRS to response, complete response rates, partial response rate, early and late toxicity, and quality of life.

## 3 Results

**Figure 1:**
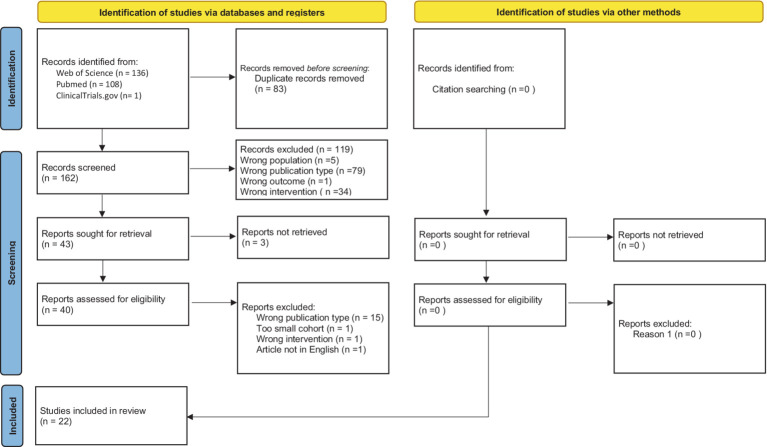
PRISMA flow chart.

During searching databases and registry process, initially 245 studies were found (136—Web of Science, 108—PubMed, and 1—Clinicaltrials.gov). Before screening, we have deleted 83 duplicates. In the next step, we excluded titles and abstracts that did not follow the inclusion and exclusion criteria (119 articles). Full texts of qualified articles were analyzed, and selection was made after which 15 studies were excluded. Finally, 22 primary studies were included in systematic review. Figure 1 shows PRISMA 2020 flowchart with screening results. List and characteristics of included studies and their outcomes are shown in Table 2. In total, 15 included studies have prospective design and 7 studies are retrospective analyses.

**Table 2:** Summary of the analysed studies.

References	Author	Year	Country	Study type	Radiotherapy Modality	No. patients	Median dose	Tremor scale	Response rate**	Improvement rate***	Time to first improvement	Adverse effects rate	Type of Adverse effects	Median follow-up (months)
(12)	Niranjan A.	2000	USA	Prospective	Gamma Knife	8	140 Gy	FTM	100%	75%	6 weeks	8% - transient	Weakness of arm and leg; dysarthria	6
(13)	Ohye C.	2005	Japan	Prospective	Gamma Knife	11	130 Gy	UPDRS	80–85%*	70%. *	6 months ^*^	N/A	N/A	24
(11)	Kondziolka D.	2008	USA	Retrospective	Gamma Knife	31	135Gy	FTM	88%	Tremor score 54% handwriting 39,2%	N/A	7,7% - mild	Hemiparesis dysphagia	36
(14)	Lim S. Y.	2010	Canada	Prospective	Gamma Knife	11	135Gy	FTMADL, UPRDS	18,2%	8,6%	N/A	16%; 11% - mild 5% - severe	Speech difficulty, hemiparesis; mild (nondisabling) lip and finger numbness	19.2
(15)	Young R.	2010	USA	Prospective	Gamma Knife	161	147Gy	FTM	81% in drawing, 77% in writing	Drawing 51% Writing 58% both 51%	N/A	5,8% - any 3,4%- severe, transient 2,4%- mild, transient	Sensory loss; motor impairment; speech disturbances; hemiparesis dysarthria,	44
(16)	Ohye C.	2011	Japan	Prospective	Gamma Knife	13	130 Gy	UPDRS	96,2% *	50%	>3 months	1,3% - transient mild	N/A	24
(17)	Kooshkabadi A.	2013	USA	Prospective	Gamma Knife	48	140 Gy	FTM	42%	Writing 48,1% tremor score 45,5% The water-drinking score 45,2%.	N/A	* 4,65% - any	Hemiparesis; dysphagia; perioral burning sensation with left-sided facial numbness.	11.5
(18)	Loiselle C.	2014	Sweden	Retrospective	N/A	99	130 Gy	FTM	77% drawing 81% writing	Spiral drawing 59,8% handwriting 69,7%	N/A	7%- any 1%- transient	Numbness, weakness, speech difficulty	10
(19)	Witjas T.	2015	France	Prospective	Gamma Knife	36	130 Gy	FTM ADL	76%	Global tremor 63.40% ADL 72.2%	5.3 months	2% severe	Hemiparesis	12
(20)	Cameron B.	2017	USA	Prospective	LINAC	21	150 Gy	FTM, QOL	71%	18,8%	3 months	3,8%- severe	Hemichoreiform movements	12
(21)	Niranjan A.	2017	USA	Retrospective	Gamma Knife	73	140 Gy	FTM	93.2%	61,4%	4.0 months	3.8% - transient	Hemiparesis;facial weakness; dysphasia numbness in the contralateral hand.	28
(22)	Tuleasca C.	2017	Switzerland	Prospective	Gamma Knife	52	130 Gy	TSTH	92%	67.8%	N/A	None	N/A	12
(23)	Tuleasca C.	2017	Switzerland	Prospective	Gamma Knife	17	130 Gy	TSTH ADL	64.7%	TSTH 67.3% ADL 82.9%	N/A	N/A	N/A	12
(24)	Niranjan A.	2018	USA	Retrospective	Gamma Knife	8	130 Gy	FTM	* 1 GKT: 100% 2 GKT: 81,9%	* 1 GKT: 66,7% tremor 74,2% writing 63,9% drawing 65,7% drinking 2 GKT: 53% tremor 44,8% drawing 43,6% drinking	* 1 GKT: 3 months 2 GKT: 3 months	* 1 GKT: 9%- severe, transient 2 GKT: None	Contralateral lower-extremity hemiparesis	35
(25)	Pérez-Sánchez J. R.	2020	Spain	Retrospective	Gamma Knife	7	130 Gy	FTM, MDS-UPDRS	85%	63,5%	* 3 months.	* 23% mild or transient adverse events, or both.	Paraesthesia; minor cognitive complaints; depression	30.0
(26)	Thomas E. M.	2020	USA	Prospective	LINAC	20 *	130 Gy	FTM/ PROMIS *	93.3% *	63.6% *	0.3–15 months.	None	N/A	> 6
(27)	Khattab M.	2021	USA	Prospective	Gamma Knife	23	160 Gy	FTM, QOL	*83%	* 50%	N/A	6% - mild	Headache	12
(28)	Ochiai T.	2021	Japan	Prospective	Gamma Knife	9	130 Gy	Videotaping	77%	62%	N/A	*12% mild-to-moderate	Motor weakness; neurological defcit	24
(29)	Luo G.	2022	USA	Prospective	LINAC	23* ET i PD	152,5Gy	FTM, QUEST	82,6%*	N/A	N/A	N/A	N/A	12
(30)	Ankrah N. K.	2023	USA	Prospective	LINAC	38	135 Gy	FTM	89.7% *	43.5% *	0.3–15 months. *	2%- severe 9,5%- mild*	N/A	>6
(31)	Horisawa S.	2023	Japan	Retrospective	Gamma Knife	27	130 Gy	FTM	88,9%	Postural tremor 55.9% Handwriting 57.6% spiral drawing 50%	5.5 months	22% - all Severe- 7% 3,5% transient Mild- 15%	Hemiparesis, foot weakness, dysarthria, dysphagia, lip numbness, and finger numbness,	32.5
(32)	Tuleasca C.	2023	Switzerland	retrospective	Gamma Knife	78	130 Gy	TETRAS, ADL	67,6%	62,3%	N/A	8.9%- any 6,4%- transient	Hemiparesis	14

UPDRS/ MDS-UPDRS, Movement Disorders Society-Unified Parkinson’s Disease Rating Scale; FTM, Fahn–Tolosa–Marin clinical tremor rating scale; TETRAS, The Essential Tremor Rating Assessment Scale; ADL, Activities of Daily Life; TSTH, tremor score on the treated hand from the FTM scale; QOL, Quality of Life questionnaire; QUEST, Quality of Life in Essential Tremor Questionnaire; PROMIS, Patient-Reported Outcomes Measurement Information System; ^*^ Results of groups including ET and other type of tremor (Parkinosn, MD) patients; ^**^ Response rate–rate of the responders (any response) in the cohort; ^***^ Improvement rate–rate of the improvement of the tremor according to the evaluation methods implemented by authors.

### 3.1 Target definition

Anatomical visualization of the intermediate ventral nucleus (Vim) of the thalamus is not a trivial task. Therefore, for the targeting of the Vim, the indirect approach based on stereotactic measurements is widely used. Indirect targeting of the Vim for the procedure of radiosurgery is usually performed in several consecutive stages. The stereotactic coordinates for the target are set using the anterior commissure–posterior commissure (AC–PC) line as a reference of interest.

The authors of 22 publications that have been included in our analysis presented a (more or less detailed) description of target localizing for stereotactic radiosurgery. In most cases, the ventral intermediate nucleus target was localized using Guiot’s diagram: 25% of the AC–PC distance (plus 1-mm anterior to the PC), 2.5 mm above the AC–PC line, and 50% of the width of the third ventricle plus 11-mm lateral to the wall of the third ventricular wall (11, 12, 14, 24, 26). Chihiro et al. decided to mildly modify the target point taking into consideration that a more accurate localization of the thalamic nuclei can be specified using the ratio of the overall thalamic length instead of the conventional reference of posterior commissure. In this case, the target point was determined as 1-mm more medial and 1-mm more anterior, which should lead to more optimal target and, as a result, a better capsular and VO sparing (13).

### 3.2 Tremor evaluation criteria

Fahn–Tolosa–MarinClinical Rating Scale for Tremor (FTM) was the most common tool in the assessment of the severity of ET. This 0–4 point score system examines the tremor intensity and the disturbance of actions of writing, drawing spirals, and, optionally, drinking water or pouring liquids (12). The tremor score on the treated hand (TSTH) scale - a scoring system based on the FTM scale criteria - was used in two studies. Other scales used included Activities of Daily Living (ADL), Patient-Reported Outcome Measurement Information System (PROMIS), Essential Tremor Rating Assessment Scale (TETRAS), and Movement Disorder Society-Unified Parkinson’s Disease Rating Scale (MDS-UPDRS). Additionally, patients’ well-being was scored using Quality of Life in Essential Tremor Questionnaire (QUEST) and Quality of Life Questionnaire. Ochai replaced the rating scale system with videotaping of a tremor in the dominant upper limb with calculation of its frequency per 10 s [measured in decihertz (dHz)] as the assessment measure (17).

### 3.3 Radiation dose and modality

The majority of the presented studies performed either unilateral or bilateral SRS thalamotomy with Gamma Knife. In four trials, SRS was performed on LINAC (20, 26, 29, 30). One trial did not specify the modality used for SRS thalamotomy (18). Median dose used was 130 Gy in single fraction, although the dose range spread between 130 and 160 Gy. The study by Kondziolka and Lims relied on the 130–140 Gy dose range (11, 14), while 130–150 Gy dose was used in two other studies (12, 21). Other authors of included manuscripts used 140–160 Gy (20), 141–152 Gy (15), 145–160 Gy (29), 156–160 Gy (27), and 140 Gy (17).

In most cases, only singular, unilateral procedure is needed to achieve the therapeutic effect. Only two publications mention bilateral procedure as a part of the performed treatment (13, 15).

### 3.4 Response to SRS

The results in considered publications in a vast majority indicate significant improvement, estimating 50–60% in the postural tremor score, handwriting, drawing, and drinking. Mean improvement rate for tremor, writing, drawing, and drinking constitute 64, 58, 52, and 52%, respectively. The patients’ quality of life is increased in comparison to the pre-operation scores—57% for the ET group and 84.6% for the mixed group (25, 27). The results of the ET and Parkinson patients represent similar results (28).

Among patients with essential tremor, most studies report extremely high response rate, reaching excellent results. Median response rate (including sub-categories of writing and drawing for the same patients) constitutes of 81.8%, meanwhile median response constitutes 81%, which is considered as good is. The maximum rate of tremor reduction was depicted in the study by Niranjan et al., reaching 100% of complete tremor reduction (24). Only two authors report on the lack of response in patients, rating this subgroup as 11.1 and 15.1% (16, 31). The state of worsened intervention than before intervention was only mentioned in the study by Ohye et al. at a rate of 3.8% (16). However, the analyzed cohort was mixed, consisting mostly of patients with Parkinson disease who are more prone to developing more severe symptoms after surgery (16).

The 6-month duration was pinpointed by several authors, reaching between 25.2 and 59.3% of tremor score improvement (13, 15, 16, 27, 28). The median improvement rate at 6-month follow-up reached 49%.

During follow-up, no additional interventions were required in most of the cases due to achieved satisfactory tremor control. In the publications by Young, Loiselle, and Niranjan, secondary intervention was needed after tremor recurrence (15, 18, 24). In the study by Lim et al., two patients underwent open surgery due to treatment failure 31 and 24 months after the initial procedure (14). Young et al. reported that nine patients needed further treatment due to increase in tremor symptoms after SRS. Those patients underwent DBS or RFT procedures due to the recurrence of symptoms (15).

### 3.5 Time to response

Median onset of the tremor decrease falls within 3 months, spanning from 6 weeks to 6 months. The post-operative 6-month mark has been pinpointed by Khattab et al. as the period of the maximum benefit for the patient, although the tremor reduction was found to increase with time in other studies (16, 27, 28).

### 3.6 Follow-up

Follow-up periods varied depending on the study type, if it was either prospective, planned, clinical trial, or retrospective. The time frame varied between mean 6 months and mean 44 months, with individual scores reaching up to 152 months (21). The mean follow-up period of all publications is 14 months.

### 3.7 Adverse effects and safety profile

The rate of adverse effects (AEs) in publications varies between 0 and 23%, with median of 7.85%. Among all analyzed studies, out of 814 patients evaluated, only 58 experienced any adverse effects (7,12%). Most of the reported adverse effects are mild and transient. Severe AEs were described in two cases. They presented as complete steroid-irresponsive hemiparesis and chronic encapsulated expanding hematoma (CEEH), resulting in dysarthria, dysphagia, and hemibody numbness. Severe dysphagia in second patient lead to death because of aspiration pneumonia 60 months after GKT (24, 31). Limb weakness, numbness, mild and transient hemiparesis and speech disturbances including dysarthria belonged to the most frequent adverse effects reported in the post-SRS patients [12, 14, 15-, 22, 25, 29, 31–33]. Other reported ailment include headaches, depression, minor cognitive deficits, sensory loss, and hemichoreiform movements (20, 25, 27, 28). Tuleasca et al. reported a correlation between BED and the severity of adverse effects (32). Permanent adverse effects were found in 15 patients including contralateral weakness, numbness, and speech disturbances. In 6 of those cases, symptoms improved after 12 to18 months. Two cases were strictly sensory in nature and of no functional consequence. One was a cause of CEEH resulted in patient death (15, 18, 31). The detailed data about AE are presented in Table 2.

## 4 Discussion

### 4.1 Comparison of the effectiveness of SRS, DBS, and MRgFUS

DBS is currently the most commonly used surgical method for treating ET, yet its invasive nature remains a significant drawback. In contrast, FUS targeting the Vim offers similar efficacy without being invasive, which becomes particularly relevant, considering the risks of brain structure damage and complications associated with invasive procedures. While FUS, such as RF and GK thalamotomy, also creates permanent lesions, its non-invasive approach may be advantageous in managing bilateral symptoms, potentially reducing the risk of complications linked to invasive treatments. Since bilateral thalamotomy is generally avoided, direct comparisons of DBS with lesion treatment procedures are more relevant to unilateral treatments. These procedures appear to have similar improvement rates, but a formal comparison is needed. While patients often adapt to DBS therapy, there are reports of early relapses following FUS surgery. The benefits of SRS are not fully predictable, as many effects develop over months. Each procedure has its own advantages and limitations, which must be considered for optimal ET treatment results (33). These findings corroborate those of other studies (34, 35).

Despite comparisons between the efficacy and cost-effectiveness of SRS and FUS, it seems crucial to consider all the biological effects of each procedure deeply, especially in the context of combined treatment. Researchers suggest that these techniques are not necessarily competing. FUS, combined with radiotherapy, particularly in malignant disease treatment, is a common scenario. Here, FUS can initially remove the main tumor mass for immediate symptom relief, followed by irradiation of the surrounding BED to reduce local and regional recurrence. Conversely, initial irradiation can damage the ability of the cells to reproduce, followed by FUS to reduce the main tumor volume (36).

A study comparing the cost-effectiveness and efficiency of the different surgical methods of tremor treatment found SRS comparably cost-effective to MRgFUS and less expensive than DBS. However, if MRgFUS is less effective or more costly than estimated, SRS may be more cost-effective. This likelihood increases considering the longer duration and higher skill requirement for MRgFUS, potentially making SRS a more prevalent treatment for drug-resistant intractable tremor (37) (see Table 3).

**Table 3:** Comparison of the efficacy, safety, and cost-effectiveness of the different thalamotomy methods.

	Stereotacitc radiosurgery	Deep brain stimulation (39)	Focused Ultrasound (40)	Radiofrequency thalatomy (41)
Rate of tremor control at 12 months	50–60%	60%	35–75%	40–64.4%^*^
Median adverse effects rate	7,85%	50–100%	36%	58–70%
Cost-driving factor	Replacement costs of the radioactive sources every 5 years	Costs of the stimulator placement and management	Restricted to one approved indication	low cost of medical supplies

^*^40% after first thalatomy at 21.3 months and 64.4% after second (other side) thalatomy at 29.3 months.

SRS appears to be more advantageous than DBS procedure in the initial ET treatment. DBS seems to bear a great potential in case of unsuccessful SRS thalamotomy. DBS performed on the patients previously treated with radiosurgery indicate potential and effectiveness of such treatment modality after recurrence of symptoms. In the study by Lim et al., one patient with PD tremor remained unresponsive to the SRS and underwent DBS on the same side. During 4 months of follow-up, patient demonstrated 80% tremor reduction (14). Similarly Tuleasca et al. report a clinical case of a patient with ET treated initially with SRS and followed-up with bilateral DBS after the SRS failure. The initial response to SRS within 6 months was excellent, although patient further relapsed after 20 months. DBS procedure resulted in immediate and complete bilateral clinical alleviation lasting for 31 months. The clinical evidence and MRI examinations suggest the lasting effect of the SRS on the structure and functionality of the affected areas. The post-SRS reorganization initiates the potential for better DBS response (38).

### 4.2 Pathophysiology and response prediction

Tuleasca et al. have utilized the results from ET treatment with SRS to explore the pathophysiology of diseases in numerous publications. They propose that components of the visual system might significantly contribute to tremor onset (varying for the finger, hand, and head tremor) and its inhibition following interventions such as SRS-T (22, 23, 42–44). In 2017 study, Tuleasca indicated right visual association area as the tremor alleviation predictor with Brodmann area (BA) 18 as the only statistically significant cluster. High pretherapeutic gray matter density also correlated with better TSTH improvement (22).

The majority of the research do not associate any form of response pattern or radiological imagining results with the clinical outcome (13, 16, 19, 23, 26, 28, 31). Linear contrasting of the border between the thalamus and the internal capsule adjacent to the lesion site was found in patients with higher symptom improvement rate in comparison to the other individuals (28). Most hyperresponders are characterized by more pronounced lesions, often times accompanied by edema. Clinical outcome of this imagining is in most cases linked with severe adverse effects, including hemiparesis (11, 12, 14, 19, 21, 24, 28, 29, 31).

Luo et al. research found the radiation dose coverage to 0.1 cm^3^ of the autocontoured VIM structure as significantly higher in responders than nonresponders and a trendfor superior coverage of the autocontoured VIM at 20-Gy isodose level in the responders versus nonresponders (29). The center of the lesion was found 0.5 ± 0.1 mm laterally to the VIM in responders in comparison to 1.1 ± 0.5 mm in non-responders, associating patient’s response with the medial–lateral position (*p* < 0,019) (29). This finding suggests that the optimal isocenter position for clinical response is 0.7–0.9 mm lateral to the geometric center of the Vim with automatic contouring (29).

Diffusion tensor imaging fiber tracking constitutes a new potential treatment isocenter localization technique, enabling localization of the dentato-rubro-thalamic tract fibers, involved in the tremor etiopathology, which potentially intersects with the target. This solution is highly promising for developing patients-specific target network; however, it is greatly limited due to the equipment and software availability (29, 45).

### 4.3 BED in the SRS treatment

There is ongoing discussion among professionals about what dose should be administered to achieve a high clinical response rate and, at same time, low rates of radiation side effects. Modern research shows that there is a significant correlation involving dose-administered and BED (biologically effective dose) with rates of response and potential ARE. It was shown that the α/β ratio for normal brain is between 2 and 3 Gy (46, 47). Tuleasca et al. made an assumption that the α/β ratio for normal brain is 2.47 Gy. In their study, it was shown that with BED between 4,300 and 4,500 Gy_2.47_, optimal response rates limiting ARE can be achieved (32).

## 5 Conclusion

The results obtained confirm the high efficacy and safety of the SRS procedure in treating drug-resistant intention tremor. A significant proportion of patients experienced a clinically meaningful reduction in tremor, following SRS with the newly developed SRS GK technology. After SRS, a considerable number of patients showed improvements in activities such as tremor, writing, drawing, and drinking. Subsequent studies underscore the enhanced safety of SRS at certain therapeutic doses (up to 140 Gy), reflecting progress compared with earlier years when higher doses (160–180 Gy) were associated with more severe complications. The method also stands out for its favorable balance of efficiency and cost, making it a competitive alternative to MRgFUS and DBS. Ongoing research is crucial to refine patient selection criteria for this procedure and further improve the effectiveness of the technique.

## Data availability statement

The original contributions presented in the study are included in the article/supplementary material, further inquiries can be directed to the corresponding author.

## Author contributions

MB: Conceptualization, Data curation, Formal analysis, Investigation, Methodology, Project administration, Resources, Supervision, Validation, Visualization, Writing – original draft, Writing – review & editing. KS: Conceptualization, Funding acquisition, Investigation, Methodology, Project administration, Resources, Supervision, Validation, Visualization, Writing – original draft, Writing – review & editing. SS: Conceptualization, Investigation, Methodology, Project administration, Resources, Software, Supervision, Validation, Visualization, Writing – original draft, Writing – review & editing. ARu: Conceptualization, Data curation, Formal analysis, Investigation, Methodology, Resources, Software, Supervision, Visualization, Writing – original draft, Writing – review & editing. NK: Formal analysis, Funding acquisition, Investigation, Methodology, Project administration, Resources, Software, Supervision, Validation, Writing – original draft, Writing – review & editing. JK: Conceptualization, Data curation, Formal analysis, Investigation, Methodology, Resources, Software, Validation, Visualization, Writing – original draft. ARo: Conceptualization, Data curation, Formal analysis, Investigation, Methodology, Resources, Software, Validation, Visualization, Writing – review & editing. WK: Conceptualization, Data curation, Formal analysis, Investigation, Methodology, Resources, Software, Validation, Visualization, Writing – review & editing. MK: Data curation, Formal analysis, Investigation, Methodology, Project administration, Supervision, Validation, Visualization, Writing – original draft, Writing – review & editing. IB: Conceptualization, Data curation, Formal analysis, Funding acquisition, Investigation, Methodology, Project administration, Software, Supervision, Visualization, Writing – original draft, Writing – review & editing. SM: Conceptualization, Data curation, Formal analysis, Funding acquisition, Project administration, Resources, Supervision, Validation, Visualization, Writing – original draft, Writing – review & editing.
